# Patients with Systemic Lupus Erythematosus Show Increased Levels and Defective Function of CD69^+^ T Regulatory Cells

**DOI:** 10.1155/2017/2513829

**Published:** 2017-09-06

**Authors:** Marlen Vitales-Noyola, Brenda Oceguera-Maldonado, Perla Niño-Moreno, Nubia Baltazar-Benítez, Lourdes Baranda, Esther Layseca-Espinosa, Carlos Abud-Mendoza, Roberto González-Amaro

**Affiliations:** ^1^Department of Immunology, Faculty of Medicine, UASLP, San Luis Potosí, SLP, Mexico; ^2^Research Center for Health Sciences and Biomedicine, UASLP, San Luis Potosí, SLP, Mexico; ^3^Laboratory of Genetics and Molecular Diagnostics, Faculty of Chemical Sciences, UASLP, San Luis Potosí, SLP, Mexico; ^4^Regional Unit of Rheumatology and Osteoporosis, Hospital Central Dr. Ignacio Morones Prieto, San Luis Potosí, SLP, Mexico

## Abstract

T regulatory (Treg) cells have a key role in the pathogenesis of chronic inflammatory and autoimmune diseases. A CD4^+^CD69^+^ T cell subset has been described that behaves as Treg lymphocytes, exerting an important immune suppressive effect. In this study, we analyzed the levels and function of CD4^+^CD69^+^ Treg cells in patients with systemic lupus erythematosus (SLE). Blood samples were obtained from 22 patients with SLE and 25 healthy subjects. Levels of CD4^+^CD69^+^ Treg cells were analyzed by multiparametric flow cytometry, and their function was measured by an assay of suppression of lymphocyte activation and through the inhibition of cytokine synthesis. We found an increased percent of CD4^+^CD25^var^CD69^+^TGF-*β*^+^IL-10^+^Foxp3^−^ lymphocytes in patients with SLE compared to controls. In addition, a significant diminution in the suppressive effect of these cells on the activation of autologous T lymphocytes was observed in most patients with SLE. Accordingly, CD69^+^ Treg cells from SLE patients showed a defective capability to inhibit the release of IL-2, IL-6, IL-10, and IL-17A by autologous lymphocytes. Our findings suggest that while CD4^+^CD69^+^ Treg lymphocyte levels are increased in SLE patients, these cells are apparently unable to contribute to the downmodulation of the autoimmune response and the tissue damage seen in this condition.

## 1. Introduction

Systemic lupus erythematosus (SLE) is a chronic autoimmune disorder characterized by loss of tolerance to self-antigens and synthesis of many different auto-antibodies with the involvement of multiple organ systems, including the skin, kidney, blood vessels, and central nervous system [1, 2]. The pathogenesis of this condition is complex, and the loss of tolerance to self-antigens observed in these patients is a consequence of multiple genetic risk factors, environmental influences, and defects in immune regulatory mechanisms, among others [1, 2].

Different mechanisms that are able to downregulate the immune response have been described, including the T regulatory (Treg) cells [3]. These cells are able to suppress the activation and proliferation of effector lymphocytes, exerting thus a key role in the pathogenesis of autoimmune and chronic inflammatory conditions [3]. Several subtypes of Treg lymphocytes have been described. Natural T regulatory (nTreg) cells show a characteristic phenotype (CD4^+^CD25^high^Foxp3^+^) and exert their suppressive effect on effector T cells through different mechanisms, including the synthesis of immune modulatory cytokines (TGF-*β*, IL-10, and IL-35) [3–7]. These Foxp3^+^ regulatory cells can be originated directly from the thymus (tTreg cells) or in the periphery (pTreg cells), by the differentiation of conventional naïve CD4^+^ cells. In addition, Treg cells with this phenotype can be generated in vitro (induced or iTreg cells) [3]. These cells have a relevant role in autoimmune diseases, and their congenital deficiency (due to mutations in the FOXP3 gene) is associated with the IPEX syndrome, characterized by immune dysregulation, autoimmune polyendocrinopathy, and inflammatory enteropathy [8]. As expected, different alterations of CD4^+^CD25^high^Foxp3^+^ have been reported in patients with SLE [9].

Other regulatory lymphocyte subsets have been described, including the T regulatory type-1 (Tr1) cells, which are generated in the periphery and mediate their suppressive effect mainly by the synthesis of IL-10 [10]. These cells do not express Foxp3 nor show a constitutive expression of CD25 but exhibit a characteristic phenotype (CD4^+^CD223^+^CD49b^high^) [11]. Additional Treg cell subsets such as CD8^+^Foxp3^+^ lymphocytes, Th3 cells, and Tr35 lymphocytes have been described, but they are less well characterized and their possible role in the pathogenesis of autoimmune diseases remains to be elucidated [7, 12].

Although CD69 was originally described as an inducible activation marker of T cells [13], a subset of CD4^+^ Treg lymphocytes characterized by the constitutive expression of CD69 has been described in both mice and humans [14]. In this regard, it has been reported that CD4^+^CD69^+^Foxp3^−^TGF-*β*^+^ cells with a variable expression of the alpha chain of the IL-2 receptor (CD25) are detected in peripheral blood and different lymphoid tissues from healthy subjects [14]. In mice, these cells exert an important suppressive role on the immune response, and in healthy humans, we have recently corroborated their presence in peripheral blood and their relevant *in vitro* suppressive effect on the activation of autologous effector T cells [15]. Furthermore, the analyses of the frequency and function of CD69^+^ Treg cells in patients with autoimmune thyroid diseases and individuals with chronic periodontitis have showed abnormal levels of these cells in peripheral blood and affected tissues as well as a defective regulatory function [16, 17]. In addition, a possible role of CD4^+^CD69^+^ Treg cells in patients with liver carcinoma has been reported [18].

NKG2D is an activating receptor expressed by most NK cells and some subsets of T lymphocytes. This molecule is a lectin-like type 2 transmembrane receptor that through its association with the DAP10 adapter molecule, it is able to generate activation signals [19]. Aside from its functional role in NK lymphocytes, it has been reported that CD4^+^NKG2D^+^ T cells exert an important immunosuppressive activity, which is apparently mediated by TGF-*β* and IL-10 [20]. In this regard, levels of CD4^+^NKG2D^+^ cells have been found to correlate inversely with disease activity in patients with systemic lupus erythematosus, although their suppressive function is apparently preserved [20]. In addition, we have recently observed that in healthy individuals, a variable proportion of CD4^+^CD69^+^ Treg cells express NKG2D, indicating an overlap between CD4^+^NKG2D^+^ and CD4^+^CD69^+^ T regulatory lymphocytes [15].

In this study, we analyzed the frequency and function of CD69^+^/NKG2D^+^ Treg cells in the peripheral blood from patients with SLE. Our data suggest that these cells seem to participate in the complex pathogenesis of this autoimmune condition.

## 2. Materials and Methods

### 2.1. Patients and Healthy Subjects

Twenty-seven patients with SLE according to the diagnostic criteria of the American College of Rheumatology were studied. Most patients were female (91%), and their mean age was 34.2 years (range 18–60 years). Most patients were receiving low-dose methotrexate (86%), prednisone (10–40 mg/day, 72%), and leflunomide (77%), but no patients under therapy with biological agents were included in the study. Eighteen patients were considered to have active disease (SLEDAI > 4.0) and nine have inactive disease (SLEDAI ≤ 4.0). No critically ill patients or with renal failure were included. Thirty healthy subjects with age and gender similar to the patients were also studied; the mean age was 36.1 years and most of them were female (95%). The Bioethical Committee of the Hospital Central Dr. Ignacio Morones Prieto approved this study, and a signed informed consent was obtained from all patients and controls.

### 2.2. Flow Cytometry Analysis

Peripheral blood mononuclear cells (PBMC) of patients and control subjects were isolated by Ficoll-Hypaque (GE Healthcare, Pittsburgh, PA) density-gradient centrifugation, and cellular viability was evaluated by trypan blue staining and it was always higher than 95%. Mononuclear cells were stained for 30 minutes in darkness at 4°C with the following monoclonal antibodies (mAbs): CD4-FITC (eBioscience, San Diego, CA) or CD4-APC/Cy7 (BioLegend, San Diego, CA), CD25-APC/Cy7 (Becton-Dickinson, Franklin Lakes, NJ), NKG2D-FITC (eBioscience), antilatency-associated peptide (LAP, a surrogate marker for TGF-*β*)-PerCp/Cy5.5 (BioLegend), and CD69-APC (eBioscience). Then, cells were washed and fixed and permeabilized with the Foxp3 Fix/Perm kit (eBioscience) for 30 minutes. Subsequently, mononuclear cells were stained with mAbs against IL-10 (PE) (BioLegend) and Foxp3 (PE/Cy7) (eBioscience). Doublet discrimination was performed by analyzing FSC-A versus FSC-W dot plots from the lymphocyte gate. In all cases, at least 1 × 10^6^ events were analyzed, and gates were set up by using fluorescence minus one controls and labeled mAb isotype controls. CD4^+^CD69^+^ and CD4^+^NKG2D^+^ cells were analyzed separately, and data were acquired in FACSCanto II flow cytometer (Becton Dickinson) and analyzed using the Flow Jo software v10 (Tree Star Inc., Ashland, OR).

### 2.3. Functional Analysis of CD69^+^Treg Cells

The suppressive function of CD69^+^ Treg cells was assessed by an assay of inhibition of cell activation, which compares the expression of the activation marker CD40L (CD154) by PBMC with or without the presence of CD69^+^ cells [21]. Briefly, PBMC of SLE patients and healthy controls were depleted or not from CD69^+^ cells by negative selection with an anti-human CD69 mAb (eBioscience), rat anti-mouse IgG MicroBeads (Miltenyi Biotec, Bergisch Gladbach, Germany), and MACS LD columns (Miltenyi). Then, PBMC (depleted and nondepleted of CD69^+^ cells) were incubated in 24-well plates (Costar, Corning, NY) precoated with an anti-CD3 (OKT3 clone, 5.0 *μ*g/ml) and an anti-CD28 (clone 28.2, 5.0 *μ*g/ml) for 7 hours at 37°C with 5% CO_2_, in the presence of an anti-CD40L/PE mAb (BD Pharmigen, San Jose, CA, USA). Finally, cells were washed and analyzed for CD40L expression in a FACSCanto II flow cytometer (Becton Dickinson), using the Flow Jo software (Tree Star).

### 2.4. Inhibition of Cytokine Release by CD69^+^ Treg Cells

The suppressive effect of CD69^+^ Treg lymphocytes on the release of cytokines by autologous cells was assayed in PBMC cultures depleted or not of CD69^+^ cells, as stated above. In this case, cells were cultured for 24 h, and at the end of incubation, supernatants were obtained and the concentration of IL-2, IL-6, IL-10, IL-17, interferon- (IFN-) *γ*, and tumor necrosis factor- (TNF-) *α* was determined by a Cytometric Bead Array (BD Biosciences). Data were acquired in an Accuri C6 cytometer (BD Biosciences) and analyzed with the software FCAP Array v3.01.

### 2.5. Statistical Analysis

Data with normal distribution were represented as the arithmetic mean and SD, and data with a non-Gaussian distribution were represented as the median and interquartile range. Analysis of 2 groups was performed with the Mann–Whitney *U* test and comparisons of 3 groups with the Kruskal-Wallis sum rank test. Data were analyzed using the Graph Pad Prism 5 software, and *p* values < 0.05 were considered as significant.

## 3. Results

### 3.1. Increased Levels of CD69^+^/NKG2D^+^ Treg Cells in Patients with SLE

**Figure 1:**
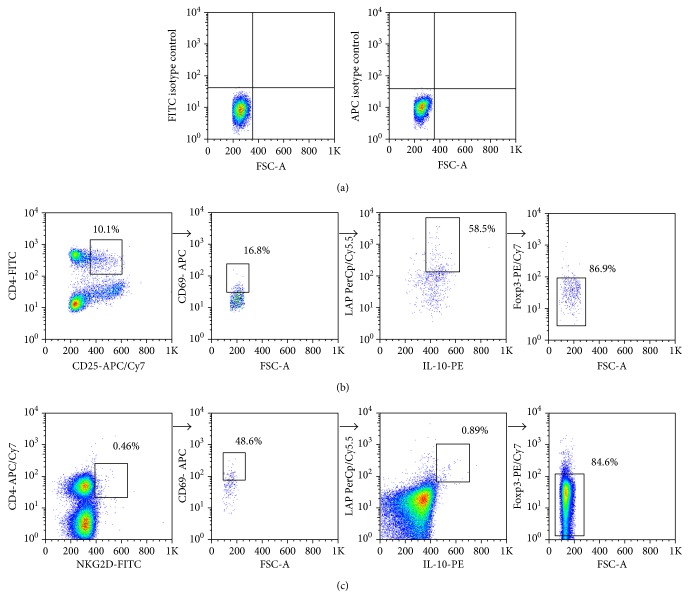
Flow cytometry strategy for the analysis of peripheral blood CD69^+^ Treg lymphocytes. Mononuclear cells were isolated from blood samples and labeled with mAbs against CD4, CD25, CD69, and LAP (TGF-*β*). Then, cells were fixed, permeabilized, and stained for IL-10 and Foxp3. (a) Dot plots of FITC and APC isotype antibody controls. (b) Strategy for the analysis of CD69^+^ Treg cells. Lymphocytes were gated on the basis of their size and complexity (not shown) and then analyzed for the expression of CD4 and CD25, CD69, LAP and IL-10, and finally for Foxp3. Results were expressed as the percent of CD4^+^CD25^var^CD69^+^LAP^+^IL-10^+^Foxp3^−^ cells, referred to mononuclear cells. (c) Strategy for the analysis of CD69^+^NKG2D^+^ Treg cells. Lymphocytes were gated on the basis of their size and complexity (not shown) and then analyzed for the expression of CD4 and NKG2D, CD69, LAP and IL-10, and finally for Foxp3. Results were expressed as the percent of CD4^+^NKG2D^+^CD69^+^LAP^+^IL-10^+^Foxp3^−^ cells, referred to total lymphocytes. Numbers correspond to the percent of cells in the highlighted gate, referred to the total of events in the previous gate. Data correspond to a blood sample from a representative patient with SLE. In all cases, at least 1 × 10^6^ events were analyzed, and gates were set up by using fluorescence minus one controls.

**Figure 2:**
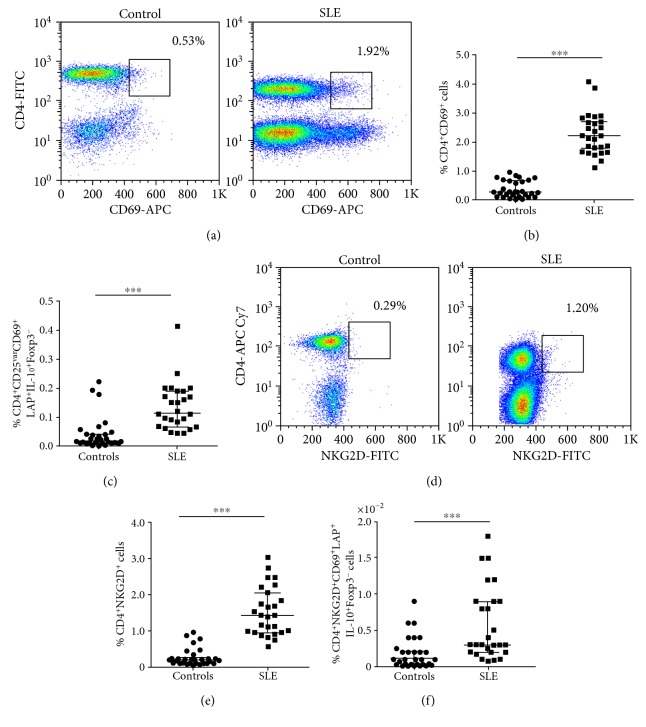
Levels of CD69^+^ Treg cells in peripheral blood from patients with SLE and healthy controls. The frequency of CD69^+^ Treg cells was determined in freshly isolated PBMC from patients with SLE and healthy controls by multiparametric flow cytometry, as indicated in Materials and Methods. (a) Representative dot plots of a CD4^+^CD69^+^ cells from a healthy control and a patient with SLE. (b) Percent of CD4^+^CD69^+^ cells. (c) Percent of CD4^+^CD25^var^CD69^+^LAP^+^IL-10^+^Foxp3^−^ cells. (d) Representative dot plots of CD4^+^NKG2D^+^ cells from a control and patient with SLE. (e) Levels of CD4^+^NKG2D^+^ cells. (f) Frequency of CD4^+^CD69^+^NKG2D^+^LAP^+^IL-10^+^Foxp3^−^ Treg cells. In all cases, the percentages of positive cells are referred to total lymphocytes. Horizontal lines correspond to the median and interquartile range. ^∗∗∗^*p* < 0.05.

**Figure 3:**
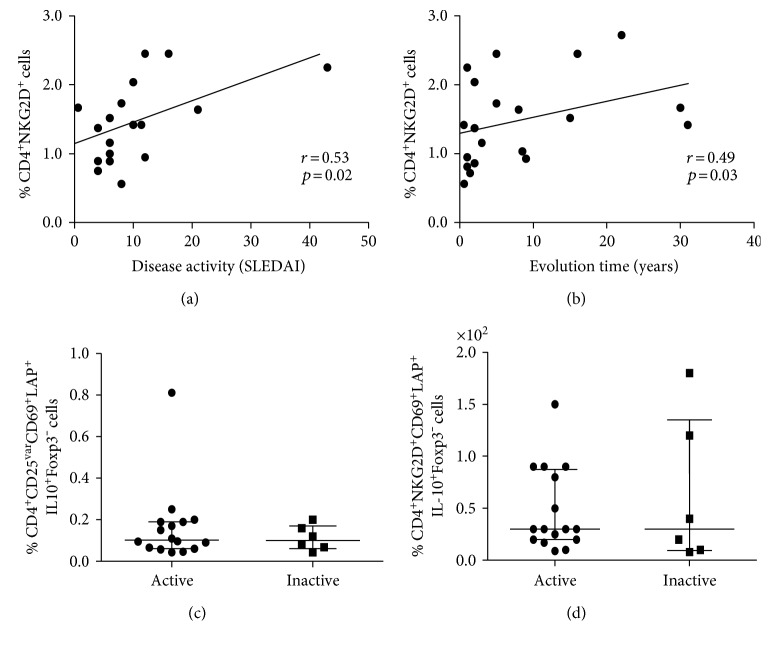
Levels of CD4^+^CD69^+^ and CD4^+^NKG2D^+^ Treg cells and clinical features of SLE patients. The frequency of CD4^+^CD69^+^ and CD4^+^NKG2D^+^ cells was determined in freshly isolated peripheral blood mononuclear cells from patients with systemic lupus erythematosus by flow cytometry, as indicated in Materials and Methods. (a, b) Correlation analysis between the levels of CD4^+^NKG2D^+^ cells and SLEDAI score or time of disease evolution in patients with SLE. *r* and *p* values (Spearman correlation test) are shown. (c, d) Levels of CD4^+^CD69^+^ and CD4^+^CD69^+^NKG2D^+^ Treg cells in the peripheral blood from SLE patients with (SLEDAI > 4.0) or without (SLEDAI ≤ 4.0) disease activity are shown.

The multiparametric flow cytometry strategy employed in this study for Treg cell analysis is shown in Figure 1. We found a significant increase in the percent of CD4^+^CD69^+^ cells in SLE patients compared to healthy subjects (Figures 2(a) and 2(b), *p* < 0.05). An additional analysis showed that SLE patients had significant increased levels of CD4^+^CD25^var^CD69^+^LAP^+^IL-10^+^Foxp3^−^ cells compared to control individuals (Figure 2(c), *p* < 0.05). However, three healthy individuals showed high levels of these CD69^+^ Treg cells (approximately 0.2%, Figure 2(c)). Moreover, we detected an increased frequency of CD4^+^NKG2D^+^ lymphocytes in blood samples from SLE patients compared to controls (Figures 2(d) and 2(e), *p* < 0.05), and the analysis of four additional markers also revealed higher levels of CD4^+^NKG2D^+^CD69^+^LAP^+^IL-10^+^Foxp3^−^ lymphocytes in SLE patients compared to healthy subjects (Figure 2(f), *p* < 0.05). A thorough analysis of possible associations between clinical data and levels of CD69^+^/NKG2D^+^ Treg cells revealed a significant correlation between the percent of CD4^+^NKG2D^+^ lymphocytes and disease activity score (SLEDAI, *r* = 0.53, *p* = 0.02, Figure 3(a)) or time of evolution of the disease (*r* = 0.049, *p* < 0.03, Figure 3(b)). However, no additional associations were detected between the levels of CD4^+^CD69^+^ or CD4^+^NKG2D^+^ lymphocytes and clinical or laboratory data (data not shown). Likewise, when the proportions of CD4^+^CD25^var^CD69^+^LAP^+^IL-10^+^Foxp3^−^ cells or CD4^+^NKG2D^+^CD69^+^LAP^+^IL-10^+^Foxp3^−^ lymphocytes were compared separately in patients with active (SLEDAI > 4.0) and inactive (SLEDAI ≤ 4.0) disease, similar values were observed (*p* > 0.05 in both cases, Figures 3(c) and 3(d)). Accordingly, nonsignificant associations were observed between SLEDAI score and the levels of CD4^+^CD69^+^ lymphocytes or CD4^+^CD25^var^CD69^+^LAP^+^IL-10^+^Foxp3^−^ (*r* = 0.32 and *r* = 0.24, resp., *p* > 0.05 in both cases, data not shown).

### 3.2. Functional Analysis of CD69^+^ T Cells

**Figure 4:**
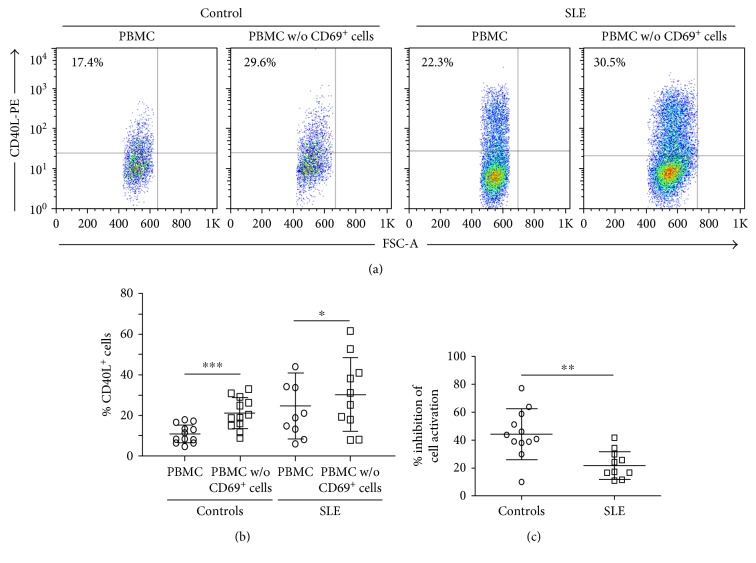
Suppressive function of CD69^+^Treg lymphocytes in patients with SLE and healthy controls. Freshly isolated PBMC from 10 patients with SLE and 12 healthy individuals were depleted or not of CD69^+^ cells and then stimulated through CD3/CD28 for 7 hours. Finally, the expression of CD40L was assessed by flow cytometry. (a) Representative dot plots of CD40L expression in cell cultures of nondepleted (PBMC) or depleted (PBMC w/o CD69^+^ cells) from CD69^+^ cells in samples from a healthy control (left) and a SLE patient (right). (b) Levels of CD40L^+^ cells in samples from healthy controls and SLE patients. (c) Percent of suppression of cell activation in samples from healthy controls and SLE patients. Data correspond to the arithmetic mean and SD. ^∗^*p* < 0.05, ^∗∗^*p* < 0.01, ^∗∗∗^*p* < 0.005.

The regulatory function of CD69^+^ T lymphocytes was analyzed by two different assays, inhibition of cell activation and suppression of cytokine release. As shown in Figures 4(a) and 4(b), in samples from healthy individuals, the activation (expression of CD40L) of PBMC stimulated through CD3/CD28 and cultured for 7 h was significantly increased when these cells were depleted of CD69^+^ lymphocytes (*p* < 0.001), revealing thus the immune suppressive effect of these cells. A similar trend was observed in blood samples from SLE patients (Figures 4(a) and 4(b)); however, in this case, the difference in CD40L expression between PBMC depleted or not of CD69^+^ cells was smaller (*p* < 0.05) compared to controls. Accordingly, when the percent of inhibition of cell activation was calculated, a significant lower suppressive capability of CD69^+^ cells from SLE patients was evident, compared to healthy subjects (*p* < 0.01, Figure 4(c)).

**Figure 5:**
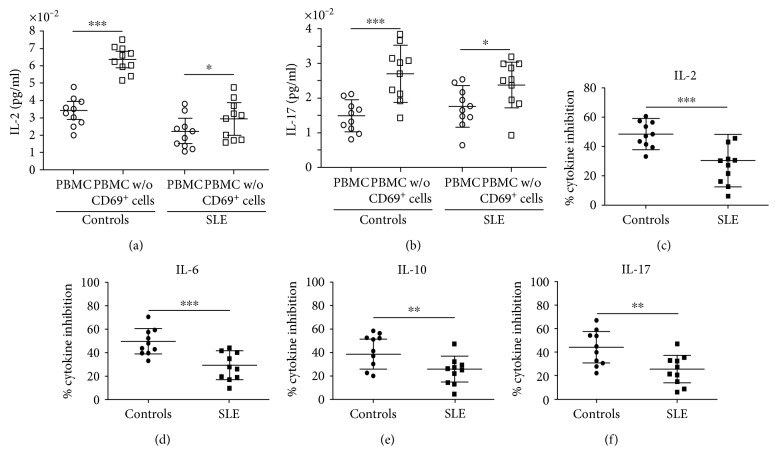
Inhibition of cytokine release by CD69^+^ Treg cells from patients with SLE and healthy controls. PBMC isolated from 10 patients with SLE and 10 healthy individuals were depleted or not of CD69^+^ cells and stimulated through CD3/CD28 for 24 hours. Then, cell culture supernatants were obtained and the concentrations of the indicated cytokines were determined by flow cytometry analysis. (a) IL-2 concentrations in PBMC culture supernatants depleted (PBMC w/o CD69^+^ cells) or not (PBMC) of CD69^+^ cells. (b) IL-17 concentrations in PBMC culture supernatants depleted (PBMC w/o CD69^+^ cells) or not (PBMC) of CD69^+^ cells. (c, d, e, f) Percentages of inhibition of cytokine release, which were calculated, as described in Materials and Methods, are shown. Data correspond to the arithmetic mean and SD. ^∗^*p* < 0.05, ^∗∗^*p* < 0.01, ^∗∗∗^*p* < 0.005.

In another set of assays, the suppressive effect of CD69^+^ Treg cells on the *in vitro* release of cytokines was assessed, by comparing the concentrations of IL-2, IL-6, IL-10, IL-17, IFN-*γ*, and TNF-*α* in culture supernatants from unfractionated PBMC and PBMC depleted of CD69^+^ lymphocytes. As shown in Figure 5, we observed a significant diminished inhibitory effect of CD69^+^ cells from blood samples of SLE patients on the release of IL-2, IL-6, IL-10, and IL-17 (*p* < 0.01 in all cases, compared to healthy subjects). Although in the case of IFN-*γ* and TNF-*α* a similar trend was observed, no significant differences were reached, likely by the limited number of samples studied (data not shown).

## 4. Discussion

Although CD69 was originally described as an inducible molecule involved in the activation of different immune cells [13], subsequent findings in CD69-deficient mice indicated that the main functional role of this receptor seems to be the downregulation of the immune response [22]. Thus, CD69 knockout mice show an enhanced severity of different models of inflammatory autoimmune diseases, including collagen-induced arthritis, asthma, myocarditis, and contact dermatitis [22–26]. Accordingly, an additional CD4^+^ Treg cell subset has been described, characterized by the constitutive expression of CD69 and the absence of the transcription factor Foxp3 or high levels of the alpha chain of the IL-2 receptor (CD25) [14, 27]. This Treg cell subset is able to exert a relevant immune suppressive effect in vitro [14, 15], and alterations in their number and function have been described in patients with autoimmune thyroid disease (mainly Graves' disease), chronic periodontitis, or hepatocellular carcinoma [16–18]. Therefore, in this study, we decided to analyze the number and function of CD69^+^ Treg cells in patients with SLE.

In this study, we observed an increased number of CD4^+^CD69^+^ lymphocytes in the peripheral blood of patients with SLE, as previously described [28]. As expected, these cells are very likely to include both, in vivo activated effector lymphocytes (bearing CD69 as an inducible marker) and Treg cells (bearing CD69 as a constitutive molecule). A subsequent flow cytometry analysis revealed that SLE patients show increased levels of CD69^+^ Treg cells (CD4^+^CD25^var^CD69^+^LAP^+^IL-10^+^Foxp3^−^) in their peripheral blood, a finding that has also been detected in other inflammatory conditions [16, 17]. Thus, although this is not an unexpected finding, the cause of this phenomenon, in patients with SLE and other conditions, remains to be elucidated. In this regard, we and others have previously observed increased levels (in peripheral blood and affected tissues) of Foxp3^+^ Treg cells in patients with inflammatory autoimmune conditions [16, 17]. However, these Treg cells seem to exert a defective immune suppressive effect, suggesting that their increased levels may be part of a failed compensatory phenomenon of the immune system triggered by the ongoing tissue damage. Moreover, it is possible that CD69^+^ Treg lymphocytes may be part of the cellular plasticity of CD4^+^ cells similar to that observed in CD4^+^Foxp3^+^ Treg cells, resulting in lymphocytes with this regulatory phenotype but with no immune suppressive activity [29]. In this regard, it is of interest that we detected that three healthy females included in the study showed high levels of CD69^+^ Treg cells (approximately 0.2%, referred to total lymphocytes). However, in these three cases, no clinical or serological evidence of an autoimmune or chronic inflammatory disease was detected.

An enhanced number of CD4^+^NKG2D^+^ Treg cells were also detected in the peripheral blood of SLE patients included in this study. In this regard, it has been reported that patients with juvenile SLE show an inverse correlation between blood levels of CD4^+^NKG2D^+^ Treg cells and disease activity (SLEDAI), with no apparent defect in the immune suppressive activity of these cells [20]. Although the patients included in our study also showed increased levels of CD4^+^NKG2D^+^ cells, with a significant correlation with disease activity, it is evident that these lymphocytes may correspond to regulatory cells (also detected in healthy individuals) or cells with proinflammatory activity (described in patients with autoimmune inflammatory conditions). However, a subsequent analysis of these cells showed that SLE patients indeed exhibit an increased proportion of CD4^+^NKG2D^+^CD69^+^LAP^+^IL-10^+^Foxp3^−^ Treg lymphocytes; however, we did not detect a significant association between the levels of these cells with disease activity or other clinical or laboratory parameters. In this regard, we have previously observed that there is an overlap of CD69^+^ and NKG2D^+^ Treg cells [15]. Therefore, we consider of interest to further characterize the CD69^+^/NKG2D^+^ Treg lymphocytes in patients with SLE and to elucidate their possible association with clinical and laboratory parameters.

Our data on the defective functional activity of CD69^+^ Treg cells in patients with SLE suggest their possible involvement in the pathogenesis of the inflammatory and autoimmune phenomena observed in this condition. In this regard, there are several reports regarding altered numbers and defective activity of Foxp3^+^ Treg cells and other regulatory lymphocyte subsets (e.g., Tr1-like cells) in patients with SLE [9, 30]. Therefore, we consider that it is very feasible that a defective activity of CD69^+^ Treg cells may also contribute to the abnormal autoimmune reactivity that is observed in SLE.

In addition to the defective capability of CD69^+^ Treg cells from SLE patients to suppress the activation of effector autologous lymphocytes observed in this study, we also assessed their effect on the in vitro release of different cytokines. Although these assays were performed in a small number of individuals (due to the limited number of cells isolated from the blood of SLE patients), our data indicate a significant reduced capability of CD69^+^ Treg cells from SLE patients to suppress the release of IL-10. Even though this soluble mediator has been widely considered as an “anti-inflammatory cytokine,” different data indicate that IL-10 exerts a pathogenic role in SLE [31, 32]. Likewise, other cytokines analyzed in this study as IL-6 and IL-17 also participate in the pathogenesis of this condition. Thus, our data on the defective regulation of IL-6, IL-10, and IL-17 released by CD69^+^ Treg cells in SLE further support the possible involvement of these lymphocytes in the pathogenesis of inflammatory autoimmune conditions [17]. However, in the case of IL-2, it is not clear whether or not a deficient downregulation of this cytokine by CD69^+^ Treg cells may contribute to the immune dysregulation observed in SLE. In this regard, it has been described the defective production of IL-2 in patients with SLE as well as the critical role of this cytokine in the generation of Foxp3^+^ Treg cells [33, 34].

In conclusion, the altered number and defective function of CD69^+^ Treg cells observed in this study suggest that these cells are another relevant component in the intricate pathogenesis of the immune dysregulation observed in patients with SLE.
